# Supplementary material for: Splice-Junction-Based Mapping of Alternative Isoforms in the Human Proteome
Source: Cell Rep. Author manuscript; Available in PMC 2020 Jan 15. (PMC6961840; doi:10.1016/j.celrep.2019.11.026)
Supplement: 2 [file NIHMS1546469-supplement-2.zip › DF1/PXD006675/RightVentricle/RightVentricle_29_NDUFA5_DSGLVGLAVCNTPHER.pdf]

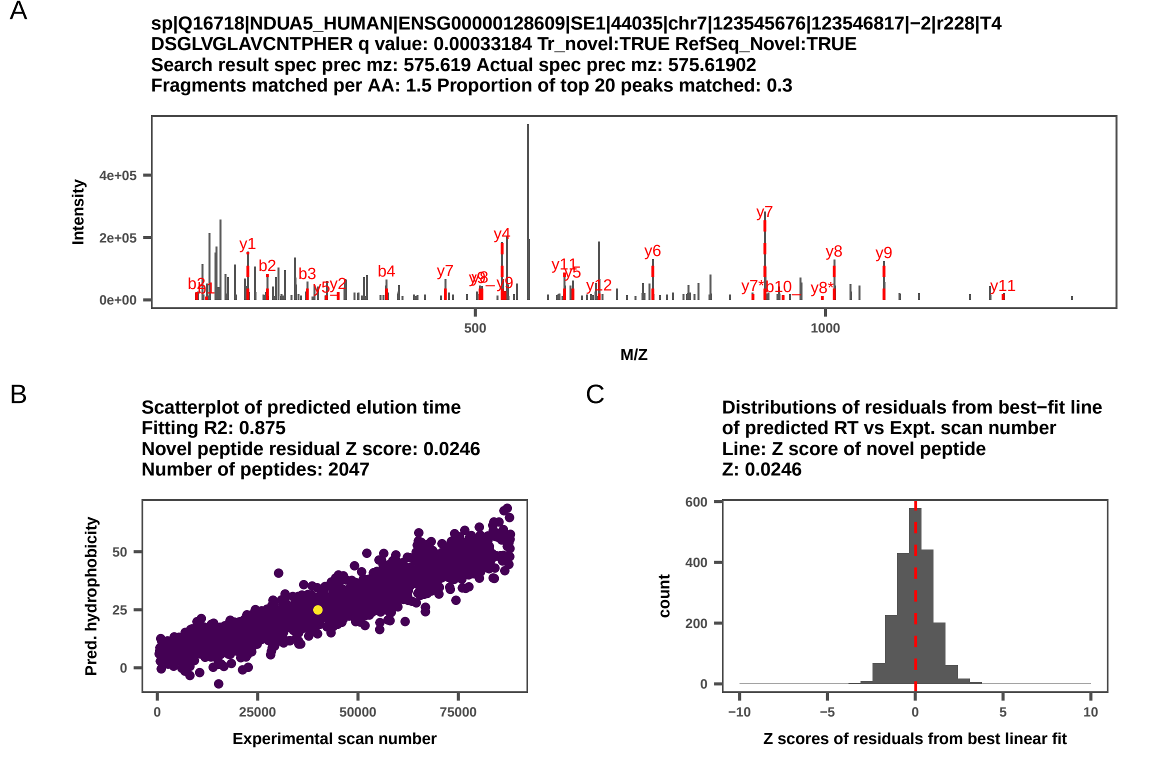
A
sp|Q16718|NDUA5_HUMAN|ENSG00000128609|SE1|44035|chr7|123545676|123546817|−2|r228|T4
DSGLVGLAVCNTPHER q value: 0.00033184 Tr_novel:TRUE RefSeq_Novel:TRUE
Search result spec prec mz: 575.619 Actual spec prec mz: 575.61902
Fragments matched per AA: 1.5 Proportion of top 20 peaks matched: 0.3
Intensity
4e+05
2e+05
0e+00
M/Z
500
1000
y1
b2
b3
b4
y7
y4
y6
y7
y8
y9
y11
y12
b10
B
Scatterplot of predicted elution time
Fitting R2: 0.875
Novel peptide residual Z score: 0.0246
Number of peptides: 2047
Pred. hydrophobicity
50
25
0
0
25000
50000
75000
Experimental scan number
C
Distributions of residuals from best−fit line
of predicted RT vs Expt. scan number
Line: Z score of novel peptide
Z: 0.0246
count
600
400
200
0
−10
−5
0
5
10
Z scores of residuals from best linear fit
